# Supplementary material for: High-Throughput Sequencing Reveals Novel microRNAs Involved in the Continuous Flowering Trait of Longan (Dimocarpus longan Lour.)
Source: Int J Mol Sci. 2022 Dec 8;23(24):15565. doi: 10.3390/ijms232415565 (PMC9779457; doi:10.3390/ijms232415565)
Supplement: Supplementary file 1 [file ijms-23-15565-s001.zip › Supplementary material.pdf]

qRT-PCR analysis of transcript levels

Table S1. The primer sequences of miRNAs and gene for qPCR analysis.

Gene name	Primer sequence
novel-miR137	CATTACTGCTGGAGCTTT
novel-miR76	GAGGATTACAATGGATTTTGT
novel-miR37	TTGATTGCGGTCTTCAAGAAG
csi-miR3954	TGGACAGAGAAATCACGGTCA
novel-miR101	TTTACTTGCAAAAGAGGAACTC
Dlo_010737.1	F: AGAAAGTGGATGGAAAGGC<br>R: TGTAGCGAGTGAAAGTGGTTAG
Dlo_009577.1	F: TAGTGGAACTGGCGAGTCTC<br>R: ATACCAAATGGCGATGCC
Dlo_027647.1	F: CGAGGATAACTTGATTCCCTG<br>R: GACTTAGATGTCCACGCTCAG
Dlo_034106.1	F: TGCTTCCCTTGGCAAAGT<br>R: AGGTTCAGATGTGGGCAA
Dlo_016181.1	F: CAGGGAGAGAATAGATGAGCA<br>R: GGGAGATACAACATTGAGCG
Dlo_025646.1	F: GACTATGGTATGTCCGTGTCAA<br>R: CATCTTTGTTCGCAGCCA
Dlo_000175.1	F: TGGCTTATGAGATTGGAAGG<br>R: AGGGACCTGGTTTAGAGGAT
Dlo_018643.1	F: GGTGGTGGGAAGAGTTATCG<br>R: TCGCCAAACAAAGCAACC
Dlo_018664.1	F: ACCACACAAGGAGATGGCA<br>R: ACTGTTGAGGAGCACCACCT
Dlo_033804.1	F: CGAGTCTCTCACCATTCAGTG<br>R: TCCGAACAACCTCATAGTCA
Dlo_029015.2	F: TTCATCCAGTTTGAGCCAG<br>R: TCTACGGGTAACATCAGGTTC
Dlo_027146.1	F: TGTCACCGAATACCACCTATG<br>R: TGCTGGAGGATTTAGATGC
Dlo_015219.1	F: CACAAATCTGATGGTGAGACC<br>R: CAGCAGTTAGAGACGCTCCT
JH-miR137-RT	GTCGTATCCAGTGCAGGGTCCGAGGTAT<br>TCGCACTGGATACGACAAAGCT
JH-miR137	F: AGACGATGCATTACTGCTGGA<br>R: CAGTGCAGGGTCCGAGGT

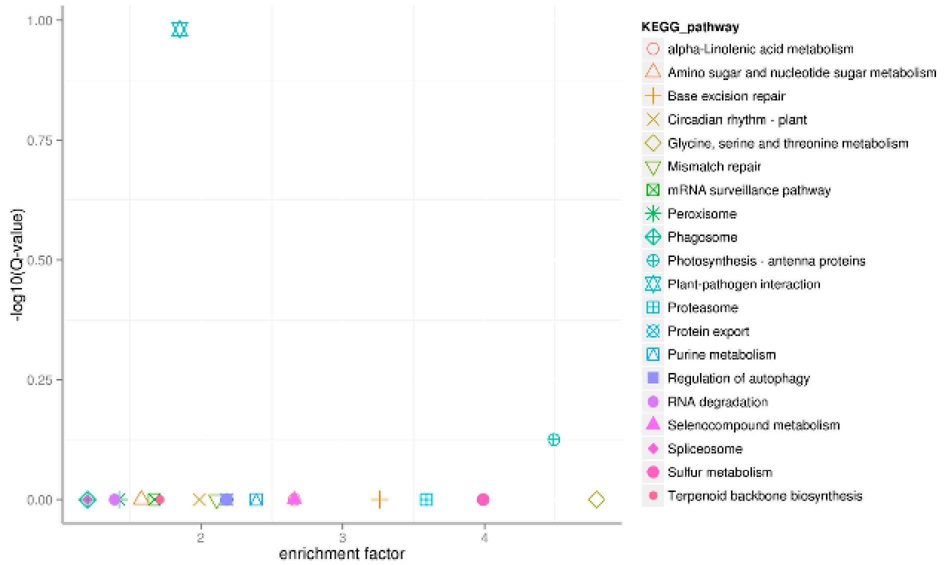

Figure S1. Differential expression miRNA target gene KEGG pathway enrichment scatter plot.

Recombinant vector construction and verification

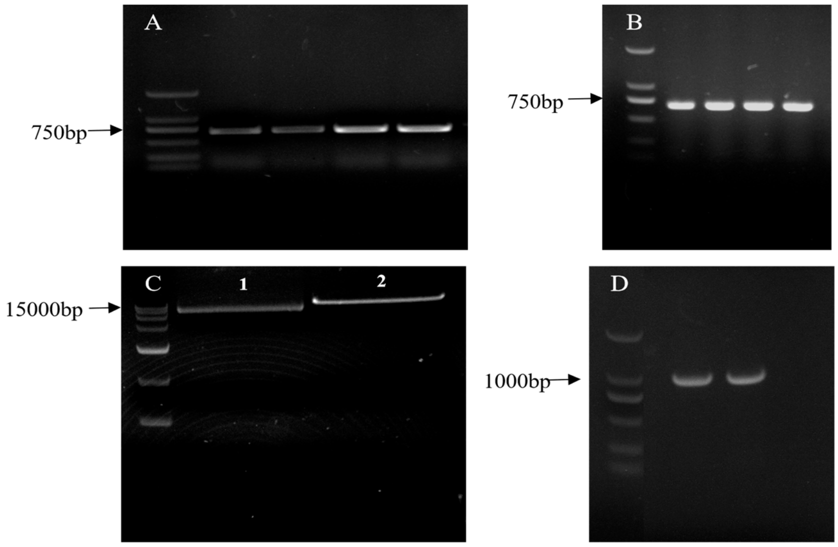

Figure S2. PCR validation of *E.coli* containing pSAK277-MIR137. (**A-B**) The amplified fragments were cloned into the pMD-18-T vector. (**C**) The pSAK277 vector was digested using restriction enzymes, which contained EcoR I and Hind III restriction sites, respectively. (**D**) The results of agarose gel electrophoresis indicated that the recombinant vector was successfully constructed.

Identification of transgenic Arabidopsis thaliana

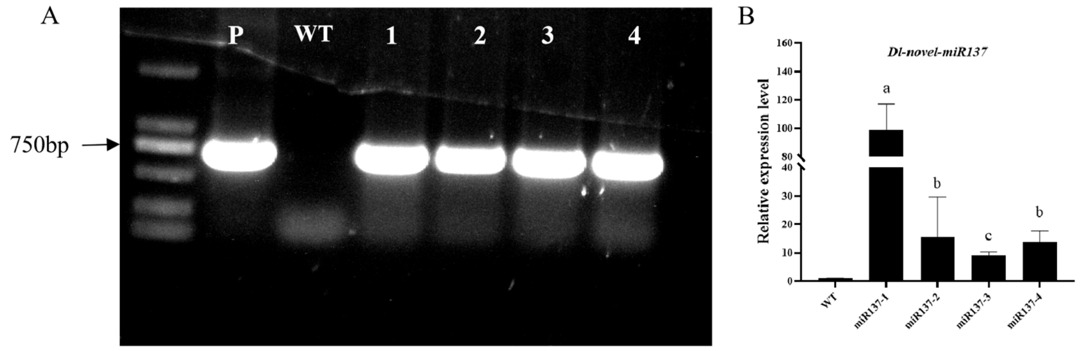

Figure S3. PCR analysis of MIR137-transgenic *Arabidopsis thaliana*. (**A**) PCR analysis of MIR137-transgenic Arabidopsis thaliana; P, pSAK277-MIR137; WT, Wild type plant; 1~4, MIR137-transgenic plant; (**B**) Expression analysis of Dl-novel-miR137 in transgenic *Arabidopsis*. Error bars show SE of the mean. Letter on the columns not the same means significantly different at (P < 0.05).
